# A Youth‐Led Evaluation of Adolescent Involvement in a Project on Development of Guidelines for Adolescent Involvement in Health Research

**DOI:** 10.1111/hex.70645

**Published:** 2026-03-25

**Authors:** Katherine Moore, Ciara Wacker, Kristin Hadfield, Azza Warraitch

**Affiliations:** ^1^ Universitat Autònoma de Barcelona (UAB) Barcelona Spain; ^2^ School of Psychology, Trinity College Dublin Dublin Ireland; ^3^ Trinity Centre for Global Health, Trinity College Dublin Dublin Ireland

**Keywords:** adolescent engagement, adolescent involvement, participatory health research, public and patient involvement

## Abstract

**Background:**

Adolescent involvement in health research has numerous benefits for the research, researchers, and the adolescents involved. However, evidence on the impacts of adolescent involvement in health research is anecdotal and subjective, relying on informal reflections rather than prospectively planned and rigorous evaluations. These limitations of the current evidence base highlight the need for more structured and prospectively planned evaluations of adolescent involvement in health research. To achieve this, we explored how adolescent co‐researchers involved in a guideline development project experienced their involvement, what facilitated their sustained participation, what outcomes resulted, and how specific engagement practices may have contributed to these outcomes.

**Methods:**

This evaluation was co‐designed and led by trained adolescent co‐researchers. Semi‐structured interviews were conducted with five adolescent co‐researchers involved in a guideline development project. Reflexive thematic analysis was conducted to analyse the data. Emerging themes were mapped onto the Youth Engagement Framework. To build a theory of change, we used the Public Involvement Impact Assessment Framework to trace how initial motivations, engagement practices, and contextual barriers contributed to observed outcomes.

**Results:**

Findings are organised around four key themes: initiators, qualities of engagement process, sustainers, and outcomes of the involvement process. Adolescent co‐researchers reported being motivated by opportunities for skill development, career growth, and roles aligned with their interests, and highlighted the role of supportive involvement practices in sustaining their involvement. They shared some predominantly positive outcomes from their involvement, including enhanced research knowledge and skills, co‐authorship opportunities, financial compensation, and strengthened relationships with the research team.

**Conclusion:**

Adolescent engagement is shaped not just by individual motivation, but also by social and institutional factors, indicating the need for system‐level changes to enable meaningful and equitable involvement. To maximise benefits, researchers should tailor involvement processes to adolescents' needs, supported by a clear theory of change.

**Public and Patient Involvement:**

This evaluation was co‐designed with adolescents and led by a trained adolescent co‐researcher. Three young people (aged 17–24) were involved at different stages of the study. One acted as a consultant during the study design phase, helping to define the evaluation framework and refine interview guides. The others were hired as co‐researchers to support data collection and analysis. The lead adolescent co‐researcher conducted interviews, proofread transcripts, supported coding and theme development, and contributed to manuscript drafting.

## 1 Introduction

Adolescent involvement in health research is grounded in Article 12 of the UN Convention on the Rights of the Child (UNCRC), which recognises young people's right to participate in decisions affecting them [1]. This principle has gained traction in adolescent health research [2, 3]. Involvement is defined as conducting research ‘with’ or ‘by’ adolescents, rather than ‘to’ or ‘for’ them [4], promoting accountability, transparency, and youth empowerment [5, 6]. Successful and meaningful adolescent involvement requires adequate planning and preparation, including ensuring mutual benefit, defining intended outcomes, and outlining methods for evaluating process and impact [7].

Research on adolescent involvement in health research has established numerous positive impacts and benefits of involvement for the research, researchers, and the adolescents involved [7]. Adolescent involvement has been reported to increase the relevance and impact of research by ensuring it addresses the specific needs and preferences of young people [4, 8]. For adolescents, involvement is associated with empowerment, enhanced wellbeing, increased knowledge and skills, as well as various other developmental, operational, and societal benefits [4, 9]. However, the current evidence on the impacts of adolescent involvement in health research is limited in several ways. Despite multiple studies reporting positive outcomes of involvement, there is a lack of evidence on the mechanisms underlying these impacts [10]. Questions remain about how adolescent involvement is beneficial, for whom it is most effective, and under what conditions these benefits are maximised [10, 11]. Furthermore, much of the evidence is anecdotal and subjective, relying on informal reflections rather than prospectively planned and rigorous evaluations [12, 13, 14]. Studies that report methods for evaluating adolescent involvement [14] often use informal approaches such as internal feedback and reflections from contributors and research teams [15, 16, 17] and activities to gather insights on adolescents' experiences [18]. The absence of structured, planned evaluations introduces a risk of biased reporting. Researchers who are strong advocates for adolescent involvement or who personally enjoy the process may report overly positive outcomes, leading to potential overreporting of positive impacts. While informal evaluation methods are valuable for gauging adolescents' satisfaction and gathering feedback to improve the involvement process, more rigorous evaluations are needed to understand the mechanisms that explain how different processes of involvement contribute to various impacts [19]. A recent review emphasised the importance of qualitative approaches and longer‐term evaluation for capturing rich insights into experiences of the involvement process and sustained impact on children and young people [20].

Another significant limitation in the current evidence on the impacts of adolescent involvement in research is the inadequate participation of adolescents in planning and evaluating their involvement, as well as a predominant focus on impacts reported from researchers' perspectives [14]. This concern has been highlighted in recent work showing that commonly used approaches to documenting adolescent involvement can privilege researcher perspectives and reproduce anecdotal accounts, particularly when adolescents are not actively involved in the design of evaluation and reporting frameworks [21]. This limitation is further reinforced by a review of existing guidelines on adolescent involvement in health research, which indicates that adolescents are rarely involved in the development of the very guidance intended to shape their participation, thereby perpetuating adult‐led assumptions about what meaningful involvement entails [22]. This imbalance emphasises the need for adolescent‐led evaluations [13] to ensure that adolescents' voices are prioritised, and the outcomes genuinely reflect the benefits and challenges they experience, rather than being shaped by adult interpretations or assumptions about perceived benefits for adolescents.

Such limitations highlight the need for more structured and prospectively planned evaluations to provide a more robust, evidence‐based understanding of the impacts of adolescent involvement in health research [13, 14, 23, 24]. To achieve this, we explored how adolescent co‐researchers involved in a guideline development project experienced their involvement, what facilitated their sustained participation, what outcomes resulted, and how specific engagement practices may have contributed to these outcomes.

## 2 Methods

This study uses a qualitative design. Ethics approval for this study was obtained from School of Psychology Ethics Committee at Trinity College Dublin (Application number: 3432). We followed the guidelines for ensuring reflexivity and rigour in qualitative research [25] and used the GRIPP‐2 reporting checklist for ensuring transparency in reporting this study [26].

### 2.1 Co‐Production of Evaluation Study

This evaluation was co‐designed with adolescents and led by a trained adolescent co‐researcher. Three young people (aged 17–24) were involved at different stages of the study. One acted as a consultant during the study design phase, helping to define the evaluation framework and refine interview guides. Two others were hired as adolescent co‐researchers to support data collection and analysis. Due to scheduling conflicts, one withdrew and was replaced by a second trained co‐researcher. The lead adolescent co‐researcher conducted interviews, proofread transcripts, supported coding and theme development, and contributed to manuscript drafting.

### 2.2 Participants and Data Collection in This Study

All seven co‐researchers involved in a previous guideline development project on adolescent involvement in health research were invited to participate via email by KM, the adolescent co‐researcher hired for this evaluation. Of the seven co‐researchers involved in the earlier guideline development project, five consented to participate in the present study. The two co‐researchers who did not participate reported time constraints and competing academic commitments as their reasons for non‐participation. Interviews were scheduled by KM at a date and time of their preference. The demographic characteristics of these co‐researchers are presented in Table 1.

**TABLE 1:** Demographic characteristics of adolescent co‐researchers.

Pseudonyms	Role in the guideline development project	Gender	Age	Education	Country of residence
Saoirse	Umbrella review: Data extraction from up to 10% of articles, narrative synthesis of umbrella review, co‐authored four manuscripts, presented review findings at a departmental seminar.	Female	24	Undergraduate psychology student	Ireland
Bella	Umbrella review: Contributed to write‐up of the introduction and methods section of a paper as a co‐author. Rapid review: Contributed to the quality assessment of all guidelines.	Female	23	Masters in social work student	Ireland
Sarah	Qualitative study: Recruited participants, co‐analysed data, co‐authored a manuscript.	Female	21	Undergraduate psychology student	Ireland
Moira	Umbrella review: Designed and co‐facilitated the participatory workshop with adolescents, contributed to write‐up of the introduction and methods section of a paper as a co‐author, co‐authored three papers, presented findings from the participatory workshop at a departmental seminar. Rapid review: Contributed to data extraction and quality assessment of all guidelines, co‐authored a manuscript. Qualitative study: Recruited participants, co‐analysed data, co‐authored a manuscript. Evaluation study: Provided input on the study design and helped develop the topic guides. Participatory workshops: Co‐designed the workshops, co‐facilitated some of the participatory workshops.	Female	22	Undergraduate psychology student	Ireland
Evan	Participatory workshop: Co‐designed and co‐facilitated a workshop to interpret the findings from the umbrella review with an adolescent advisory group.	Male	23	Undergraduate psychology student	Ireland

Semi‐structured interviews were conducted with five adolescent co‐researchers by KM using Zoom. These interviews were audio recorded with participants' consent and were transcribed using Zoom's automatic caption generation function. The interviews lasted a median duration of 32 min and ranged in duration from 26 min to 38 min. A semi‐structured interview guide ensured consistency while allowing participants to share their experiences in detail. Questions explored their roles, the process and impacts of their involvement. The transcripts were proofread, corrected for accuracy, and pseudonymised by KM.

### 2.3 Data Analysis

**FIGURE 1:**
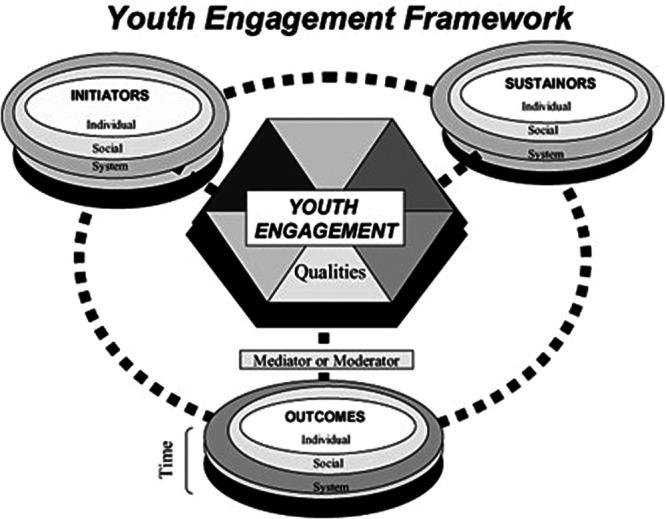
Youth engagement framework by the CEYE. *Note:* This figure shows the youth engagement model developed by and reproduced from Rose‐Krasnor [28].

The analysis followed the coding process of reflexive thematic analysis approach by Braun and Clarke [27], with themes developed by mapping the codes onto the framework described below. Reflexive thematic analysis was chosen for its emphasis on flexibility and reflexivity. The analysis process began with KM familiarising herself with the data by reviewing the transcripts during the proofreading process. She documented her initial impressions of the interviews in the form of memos, capturing key insights and reflections. Following this, KM conducted inductive coding in NVivo, applying both descriptive and interpretive codes to the data. This step was based on open‐ended coding of the data, allowing themes to emerge from the participants' narratives. KM and AW then collaborated to develop initial themes by grouping similar codes under broader categories and identifying patterns in the data. At this stage, themes were reviewed and refined by mapping them onto a theoretical framework to systematically outline the mechanisms underlying the involvement process and outcomes. Specifically, the Youth Engagement Framework developed by CEYE [28] (Figure 1) was used to categorise the codes into key areas, including individual‐, social‐, and system‐level factors that initiate and sustain adolescent involvement in research, the qualities of the involvement process, and the outcomes for adolescents.

To build a theory of change, we used the Public Involvement Impact Assessment Framework [19] to trace how initial motivations, engagement practices, and contextual barriers contributed to observed outcomes. First, the initiating factors that motivated adolescents' participation were identified and mapped alongside the engagement strategies and team values that were used to materialise these factors and sustain adolescent involvement. This process was further supplemented by identifying the practical challenges faced by the research team in implementing the engagement strategies. These layers were then systematically connected to the reported outcomes, such as increased research knowledge and skills, to illustrate how adolescents' motivations informed the use of engagement strategies and team values, how practical challenges influenced their implementation, and how these mechanisms were linked to specific outcomes. Pathways were visually represented, with solid lines indicating direct relationships (e.g., training leading to skill development) and dashed lines showing indirect associations (e.g., inclusion in the wider research group resulting in increased research knowledge).

### 2.4 Positionality Statement

The research team for this adolescent evaluation study possessed a combination of professional expertise and lived experience. KM, a master's student in education policy for global development, and CW, an undergraduate student in psychology and adolescent co‐researchers, contributed their lived experience of adolescence and their active participation in youth‐focused organisations, ensuring the study design and findings were grounded in adolescents' perspectives. AW, a PhD student, brought her expertise on the processes and outcomes of adolescent involvement and how these are affected by different factors. KH, an Associate Professor, provided critical insights into the project design based on her expertise in conducting participatory research with adolescents. To address the influence of positionalities, the team ensured adolescent‐led processes were central to the evaluation to prioritise adolescent co‐researchers' views and experiences of involvement.

## 3 Results

**FIGURE 2:**
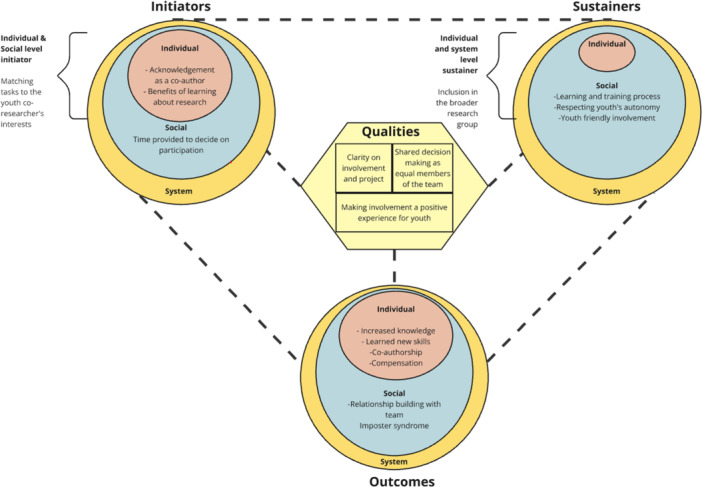
Findings mapped onto the Youth Engagement Framework.

Findings are organised around four key themes: initiators, qualities of engagement process, sustainers, and outcomes of the involvement process (Figure 2).

### 3.1 Theme 1: Initiators

This theme describes factors motivating adolescents to participate in research and barriers hindering initiation. Motivating factors emerged at individual and social levels, while barriers were primarily systemic. At the social level, providing adolescents with *adequate information and time* for voluntary participation facilitated engagement. Clear, detailed information about the project and adequate time to reflect allowed participants to consider their interest and capacity. Sarah noted, ‘*There was no pressure…I could take whatever time I needed to then give them an answer back*.’

Individual motivators included *opportunities to learn and gain research experience* that aligned with adolescents' academic and professional goals. Moira explained, ‘*I was excited about the opportunity to get experience with research, because that's something I am interested in the future*.’ Participants also saw the possibility of *acknowledgement and co‐authorship* as an opportunity to build their research portfolios, supporting their future academic and professional goals. Evan explained how this acknowledgement motivated him:

I think it was definitely in some way put forward by AW. I am not sure if it was guaranteed, because I mean, obviously it would be hard to guarantee co‐authorship until it's actually done because depending on like the amount of work and stuff like that. But it was always kind of like a motivator.

Similarly, adolescents were motivated to get involved due to the *alignment of roles* to their personal interests and skills. When tasks matched their academic backgrounds or areas they wanted to develop, participants felt they could contribute meaningfully and grow as researchers. As Saoirse noted, ‘*I was given tasks that corresponded to my knowledge and experience… while still being quite new and therefore challenging and useful for my development as a researcher*.’ Another participant similarly highlighted the relevance of their academic background to their assigned tasks, saying,

I had been learning about research, but I hadn't done a lot of research. So, it was still new in a way, and I was always interested to learn more about literature reviews, and just evidence synthesis generally so. And a lot of work that I did end up being just related to the literature review, so I think it worked as well.

(Bella)

A key systemic barrier was *limited advertisement of opportunities*, restricting diversity and equity in involvement. Evan remarked, ‘*I came across the position because I knew somebody else working on the project…if I had heard of it through the school I suppose they could reach more people*.’

### 3.2 Theme 2: Qualities of the Adolescent Engagement Process

Participants highlighted three key aspects of engagement: clarity on the involvement process, inclusion in decision‐making, and ensuring a positive experience. Adolescents valued the *clarity of the information* provided about the project and their roles, timelines, and expectations, which helped them balance involvement with other commitments. As Bella reflected, ‘*I felt like everything was explained to me… AW did a really good job orienting us to the research*.’ However, there were areas where clarity was lacking, particularly around financial compensation which was mostly linked to uncertainty amongst the research team about the possibility of securing funding. Moira shared: ‘*I was compensated for the participatory workshops and for most of it with gift vouchers, but I'm not sure if I was initially told that*.’ Another participant noted that compensation was only communicated midway through the project:

I think towards the end the researchers got more funding and then they were able to pay us at the end of it, so that was obviously a plus and felt great to like, be able to ‐ obviously everyone would love to be paid for their work, but that was a plus. It wasn't communicated at the start.

(Evan)

While initial information was clear, participants highlighted a desire for ongoing updates to understand how their work fit into the broader project. Sarah elaborated, ‘*I knew sort of what was happening… [I may have wanted] better updates, so you are aware of what's going on rather than just your own little bubble*.’

Meaningful involvement—defined as being treated as equals and *sharing decision‐making power* over the project—was a core component of adolescents' experience. Sarah explained how this dynamic was evident during the coding process:

I would say AW was really good at, especially during the, qualitative coding phase when we would have calls to go over how the other researcher and I had coded a certain passage, and AW would tell us how she had coded it as well, and if there was ever kind of like a difference in coding, we would like discuss that, and she would ask us, why we coded a certain way, and then she explained her side, and it felt like a very much, kind of we were all on a level playing field. It wasn't her being like, well, ‘I coded it like this so, this is what it is.’ … it felt very kind of even, like equal.

Adolescent co‐researchers valued having creative freedom in the engagement process. This freedom validated their contributions and reinforced their sense of ownership. Moira explained, ‘*Whenever AW made like a poster, a recruitment poster, she would allow me to… make it kind of my own*.’

Being treated as equals within the research team was also a key factor in creating positive experiences for adolescent co‐researchers. Participants reported different preferences regarding formality, with some valuing a job‐like experience while others appreciated a relaxed environment. This tension between desire for formality and informality highlights the need for a balanced approach that caters to the diverse expectations and needs of adolescent co‐researchers. Saoirse shared her experience of being engaged as a researcher:

I wanted to kind of feel like this was a job and a legitimate research role, and which it was. And it felt that way, you know? Well, when I think of youth, I suppose I think, potentially, kind of patronising? That's probably not good. And that's obviously what the project is trying not to do. But there wasn't like ‘we're going to have like free pizza, because that's what young people like’… Because this project is about youth involvement, one thing that was so clear to me all along was that like the researchers were practicing, and AW was practicing what she preaches essentially. Yeah, I just felt extremely respected as a youth co‐researcher. And there was no kind of hypocrisy at all, and it was cool reading different papers for the umbrella review, and being like ‘Yep AW is doing that, the research team is doing that.’

Moira, comparatively, appreciated the informality: ‘*AW made the meeting environment very comfortable, very casual*.’ Consistent, specific feedback from the research team was universally valued. Saoirse described how this feedback validated her work:

AW was really good for giving positive feedback. Like quite specific, when I would complete one portion of a task‐it was always clear that AW was making a conscious effort to give me positive feedback as I went along.

Appropriate workload management also contributed to positive experiences, especially for participants balancing academic commitments. Bella noted, ‘*They were very concerned about not overburdening me with anything which is fantastic*.’

While participants generally reported positive experiences, they suggested two key improvements: more interaction among adolescent co‐researchers to strengthen belonging (Saoirse: ‘*Working in the same environment and having more contact with other adolescent co‐researchers would have improved the experience’*) and simplifying payment mechanisms, as the voucher system was reported as overly complicated. This system‐level factor reflects how practical barriers, such as inefficient payment systems, can detract from participants' experiences, as highlighted by a participant:

Receiving like One4All vouchers can be a little bit complicated, like just the way… I was given like 3 different One4All vouchers, with like different bits of amounts of money on them which was just like complicated to spend. Basically, it's like a little bit more difficult to spend when you get like a photo of a One4All voucher … I think straightforward like payment is easier.

(Saoirse)

### 3.3 Theme 3: Sustainers

Adolescent co‐researchers identified social‐ and system‐level factors influencing sustained involvement: prioritising learning and training, respecting autonomy, making involvement adolescent‐friendly, and inclusion in the wider research institute. *Learning and training opportunities* facilitated continued adolescent involvement Participants' inexperience was acknowledged without being stigmatised, as Saoirse noted, ‘*As a young person, I don't have as much research experience [or]…knowledge…it was clear to me that was okay, and I was given the support I needed*.’ Another participant reflected on their experience of practicing their tasks with the research team:

Before the webinar, we held a couple of practice sessions as this is something I'm a bit less comfortable with, like speaking and public speaking. So, we kind of practiced how best to like present it naturally, not just reading off the slides. And that helped a lot with that.

(Moira)

A key aspect of the training process that adolescents identified as a facilitator to their learning was scaffolding, where adolescents were provided with practical demonstrations or examples before taking the lead themselves, with support available as needed. This kind of guidance gave participants concrete examples to follow, reducing uncertainty and ensuring that adolescents felt confident before progressing to independent work. Sarah remarked,

I think we coded, I want to say 4 transcripts, and we did them like one at a time, and AW checked in with us after the first and second one, and we went through it like slowly, step by step, so that she made sure that we like, understood everything. So, it was like kind of on the ball training, in a sense. And then, once we were like, we felt good, AW checked in with us if we were happy to code the other two like just by ourselves, which is good.

Templates and examples further facilitated learning, with one participant describing it as ‘*learning by example’* where ‘*Any task I did AW had already started doing it. So, there were some examples for me to go off*.’ *(Saoirse)*. Practice opportunities, such as preparation sessions before webinars, ensured participants felt capable of engaging with new and complex tasks.

Adolescent co‐researchers highlighted the significance of *autonomy and choice in their participation*. At the social level, autonomy was ensured through the team's commitment to offering adolescents choices about how much work they took on, which tasks they worked on, and how they participated. Sarah shared, ‘*Every new step in the project AW was always like, ‘This is what we're doing. Are you interested in it?’…I never felt pressured…I could have pulled out at any time*.’ This practice of asking for consent and gauging interest ensured participants felt in control of their involvement and created an environment where adolescents could choose to engage based on their capacity and interest. Participants appreciated the flexibility in being able to decide where they worked from, or when and how meetings were held. Evan explained, ‘*We were never forced to attend meetings or anything*.’

*Adolescent‐friendly involvement* reflected the research team's efforts to create a supportive and inclusive environment through flexibility, structured learning, and regular check‐ins. Moira noted the team's flexibility with academic commitments, ‘*AW really took into account our academic commitments…one time she wanted to hold a meeting and I said, ‘I could be there after my exam,’ and she said, “No, we'll do it next week or the week after.”’* This flexibility was not only logistical but also relational, promoting trust and a sense of agency. Participants felt empowered to communicate their limits without fear of judgement, as Bella shared, ‘*They were really flexible and understanding…it was very clear if I needed to focus more on my dissertation, I would have to take a leave, and they were completely fine with that.’* The serial structuring of tasks into manageable chunks made involvement less intimidating and more engaging. As Sarah explained,

The kind of slow staggering of work, giving like small pieces at a time made it really manageable, which was good and like that, then that lent itself to having those regular check‐ins, because it was kind of like before and after each kind of activity or like work you were doing, then there was an obvious need for a new check in, because you were getting new work or talking about the work you had just done … if you ever want an out, you know that it's like this little chunk will be done in a week or so, and then you can be like, ‘Oh, this is actually not working out for me anymore’ versus if you were just given like a big long task that seemed never ending, there wouldn't necessarily be that opportunity to kind of have a check in and say, this actually isn't working anymore.

At the systemic and social levels, adolescents reported *inclusion in the broader research group* as a key motivating factor for their continued involvement. Moira described, ‘*AW invited me to go to her supervisor's lab meetings…that made me feel really included*.’ This was similarly noted by Evan, ‘*We were added to the mailing list of the Trinity Centre for Global Health… I remember thinking, “This is so nice that I'm actually a part of this research group’”*. Participants appreciated having access to physical spaces associated with the research group, symbolising their inclusion and ensuring they had the resources needed. Bella noted, ‘*I had my own desk in their office. I had a key to go in whenever I wanted. There was always physical space for me to go to*.’

### 3.4 Theme 4: Outcomes of Involvement for Adolescent Co‐Researchers

Adolescents reported several individual outcomes including increased knowledge, skill development, co‐authorship recognition, and financial compensation. Adolescent co‐researchers reported an *increase in knowledge* about research methods, including evidence synthesis, qualitative methods such as coding, participatory approaches, and adolescent involvement in research as a right. This gain in knowledge often had broader applications in their academic and professional pursuits. One co‐researcher shared: ‘*Getting some experience with qualitative data analysis was really helpful, and that actually made me want to do my final year dissertation with a qualitative study*.’ *(Moira)*. Evan echoed this sentiment, highlighting the exposure and passion gained for participatory research methods: ‘*It taught me so much about…the whole participatory approach. I have gone on to use that in my undergrad thesis, and it's something I don't think I would have been exposed to until much later if I hadn't taken part in this project*.’

Adolescents developed transferable skills, including using research software, running workshops, recruitment, and public speaking. Evan highlighted the transferable nature of facilitation skills gained through running workshops: ‘*We were running the workshop…so I suppose that was training in itself, because it was really helpful for qualitative stuff I did later…it kind of feeds into focus groups as well*.’

*Acknowledgement as co‐authors* validated adolescents' contributions, promoted a sense of achievement, and provided concrete evidence of their involvement which aided their academic and career pathways. Bella noted, ‘*I was one of the first authors for [the paper I worked on], and I think that really helped me career‐wise later on*.’ Another co‐researcher reflected on how authorship contributed to their academic and professional goals:

Having been a coauthor and having publication credits coming out of it because, yeah, it helped me with my master's applications, and then with like, I've recently been employed, and I was able to speak a lot about my experience. And also, it just yeah, it just looks good to have some authorship to your name.

(Saoirse)

At the social level, adolescents *built meaningful relationships* with the research team based on trust and mutual respect. Researchers provided guidance on academic and career pathways, shared advice, and connected on a personal level. Saoirse reflected,

I developed a good relationship with AW, and informally she gave me some really helpful advice on my career, on PhD. That was really helpful, and also, we developed a bit of a friendship which was just nice on a personal level… at the end, AW brought me out for dinner, we had a really nice kind of chat. … just a conversation about like next steps, and yeah, that was a really nice way to finish it off. It just felt like I had made a meaningful contribution to the project.

Adolescent co‐researchers reported two negative outcomes of their involvement. Some noted while they were given opportunities to provide feedback on co‐authored papers, they did not receive input from researchers on their feedback, which was perceived as a missed learning opportunity. One participant explained, ‘*It would have been useful to get a response after [adding comments to co‐authored papers], because often I wasn't sure whether the feedback I was giving was useful*.’ (Saoirse). Some co‐researchers reported experiencing imposter syndrome, particularly when contributing to co‐authored papers or being consulted on research decisions. One participant shared, ‘*I felt like I was like underqualified to consult on’* (Evan). Another co‐researcher similarly described, ‘*Having been a co‐author on several papers I haven't always felt like I legitimately earned that credit*.’ (Saoirse)

### 3.5 Theory of Change

**FIGURE 3:**
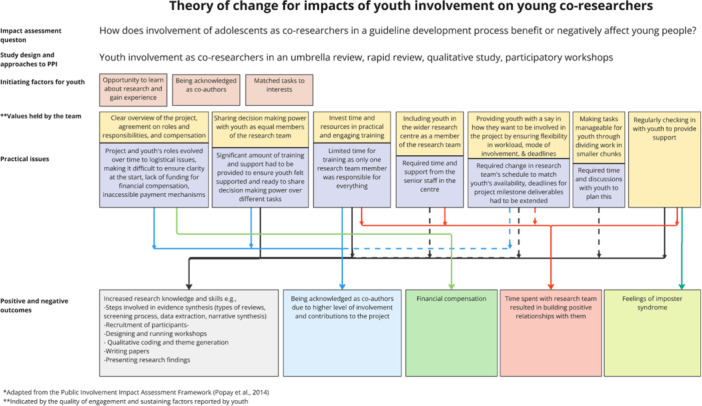
Theory of change for impacts of involvement of adolescent co‐researchers.

This study developed a theory of change, mapping how the involvement process contributed to various outcomes for adolescents. This began by identifying desired outcomes, such as learning about research and gaining practical skills, and then designing an involvement process to support these goals. The research team provided structured training and maintained regular check‐ins while positioning adolescents as equal team members with significant decision‐making power. Including adolescents in the wider research environment exposed them to journal clubs, research seminars, and other projects, broadening their understanding of research. Flexibility and autonomy supported sustained engagement and learning. The complete theory of change (Figure 3) serves as a guide to understanding how different elements of the involvement process can be intentionally designed to maximise benefits for adolescents, highlighting the interconnectedness of process design and desired outcomes.

## 4 Discussion

This evaluation highlighted that adolescent involvement, when aligned with young people's goals and supported through structured training, autonomy, and inclusion, is linked to positive and meaningful outcomes such as skill development, co‐authorship, and strengthened relationships. While most experiences were positive, challenges such as imposter syndrome and complex payment systems also emerged. The theory of change developed for adolescent involvement process identified how different initiators, sustainers, and engagement practices may have contributed to various outcomes for adolescent co‐researchers. By using a prospectively planned, adolescent‐led evaluation and applying two established frameworks, this study contributes to the evidence base on when and how adolescent involvement is beneficial [10]. By centring adolescents in the evaluation process, this study addresses a key gap: the over‐reliance on researcher perspectives in previous evaluations [14]. However, it is important to note that the findings presented in this study reflect participants' subjective experiences and perceptions of their involvement outcomes and should not be interpreted as evidence of causal relationships between specific engagement processes and reported outcomes.

Most of the findings align with prior literature on youth involvement in health research, especially regarding the value of learning opportunities, flexible structures, and meaningful participation [29, 30]. However, a notable contribution was the use of scaffolding, a step‐by‐step support strategy that decreased over time as adolescents gained confidence [31]. While hands‐on training and regular check‐ins have been cited in past research, scaffolding provides a more intentional and equity‐oriented approach to capacity‐building. It also promotes more equitable youth–adult partnerships, which have been shown to improve outcomes compared to entirely youth‐led models [32].

Similarly, the opportunity to learn about research, gain practical research experience, and develop skills adolescents could apply to their studies and other areas have been frequently reported as outcomes for adolescents in studies evaluating their involvement [29, 30, 33, 34]. However, some studies noted these benefits primarily from the researchers' perspective, with youth collaborators not always reporting training or increased knowledge and skills as significant outcomes [35]. This reinforces Forsyth et al.'s [35] argument that training may not be universally beneficial unless aligned with individual goals. In this project, adolescents' roles were shaped through early conversations about their interests, which helped ensure task relevance and more impactful experiences.

The project intentionally used a non‐hierarchical partnership model rather than a youth‐led one. While dominant models like Hart's Ladder [36], Shier's Pathways [37], the levels of youth participation framework [38] position youth leadership as the ideal, this study supports the value of shared control and flexibility [32]. Adolescents appreciated the ability to ‘participate on their own terms,’ reporting that flexible scheduling and task structures helped them stay engaged despite academic and personal commitments. Flexibility also created space for agency, an essential component of meaningful involvement. This supports critiques of hierarchical models for overlooking flexibility as a central principle in youth participation [14]. The sense of agency reported by adolescents as a result of the flexible nature of their involvement suggests partnership models are equally, if not more, valuable compared to youth‐led models. Similar reflections have been reported by young people with lived expertise involved in research, who emphasised that flexible working arrangements and suitable work environments were critical to sustaining meaningful involvement [39, 40].

Adolescents also valued being treated as legitimate researchers. They appreciated the choice to work formally or informally, depending on their preferences. For some, professionalised experiences reinforced their sense of credibility, while others valued casual interactions and approachable meeting environments. These preferences highlight the need for tailored adolescent‐friendly approaches, shaped by direct input rather than generic assumptions [30]. The findings further support pluralistic partnership models that strike a balance between adolescent autonomy and adult mentorship. This approach was particularly valuable in a research setting where adolescents were newer to research and required guidance. Shared power did not mean equal participation in every decision but rather an optimal distribution of roles based on respective strengths, a structure associated with better outcomes for young people [32, 41].

Co‐authorship emerged as a key benefit, supporting both academic and career aspirations. Although less commonly reported in prior studies, this finding may be due to the advanced education levels and research interests of the adolescents in this project. The significance adolescents attributed to co‐authorship in this study is particularly noteworthy in light of ongoing debates within the Public and Patient Involvement literature regarding whether and under what conditions participant or patient contributors should be included as authors [42]. While some scholars caution against treating co‐authorship as a default form of recompense and emphasise the importance of accountability and substantive contribution [42], the experiences reported here illustrate how co‐authorship, when grounded in meaningful research roles and supported through training and mentorship, was perceived by adolescents as both legitimate and consequential for their academic and professional trajectories. Other studies suggest that younger adolescents or those involved in youth advisory groups may not place as much value on co‐authorship [30, 34], which reinforces the need to align recognition strategies with participants' goals and capacities.

Financial compensation, while appreciated, was complicated by institutional limitations. Adolescents reported delays, overly complex voucher systems, and bureaucratic barriers in accessing pay, all of which negatively impacted their experience. These challenges mirror previous findings [43, 44], which identify youth compensation as an ongoing structural issue. Previous research on the involvement of contributors and co‐researchers has similarly reported that gift cards are perceived by researchers as a preferred option, as they are often compliant with university policies and help avoid issues around social welfare benefits [45, 46]. However, these are sometimes viewed as patronising forms of compensation by contributors [47]. On the other hand, hiring adolescent co‐researchers on the casual payroll system in this project required extensive documentation and lengthy processing times. Adolescents who chose this option faced months‐long delays and needed substantial assistance from principal investigators (KH, AW) and the centre administrator (HF) to navigate the process. Adolescents in other studies also reported the long processing times of timesheets for their casual payroll work to be challenging, as these result in delayed payments [44]. These organisational structures and policies were reported to affect the relationship between youth and research teams [44]. Organisational policies around other forms of compensation have also been reported to be inaccessible and complicated in previous studies. Other forms of payment, such as cash payments, are not allowed by most institutions, requiring research teams to opt for gift vouchers or payment through casual payroll systems [48]. These challenges reflect broader, well‐documented system‐level barriers to meaningful adolescent involvement, including rigid institutional procedures and misalignment between organisational policies and adolescents' needs [49]. These barriers underscore the need for more accessible and efficient payment mechanisms tailored to adolescent collaborators and highlight the importance of providing more support from research teams in navigating organisational payment systems.

A less‐discussed outcome of adolescent involvement, relationship‐building, emerged as especially significant. Adolescents valued the supportive, respectful, and collegial relationships formed with the research team, which contributed to a sense of belonging and confidence. While relationship‐building is often discussed as a process, it has rarely been framed as an explicit outcome [7, 29]. However, adolescents also noted challenges in building relationships with their fellow co‐researchers due to limited opportunities for collaboration and socialisation. Flexible scheduling and remote work options, while accommodating individual needs, inadvertently reduced opportunities for teamwork and interaction. For instance, adolescents often worked at different times or locations based on their commitments, which hindered peer‐to‐peer engagement.

Unlike other studies evaluating youth's experiences of being involved in research, adolescents in this project did not report a sense of making a meaningful contribution to society or positively impacting other young people's lives as an outcome of their involvement [29, 33, 34, 50]. This contrast may be due to the nature of the research topic. Previous studies reporting such outcomes often focused on areas like youth mental health or palliative care, where the societal impact was more immediate and visible to participants. In this study, the broader goal of increasing adolescent involvement in research may have felt less tangible in terms of direct societal benefit.

A key strength of this study was its adolescent‐led evaluation design, which enhanced authenticity and reduced bias. Peer‐to‐peer interviews likely supported more open and honest reflections. Several limitations of this evaluation should be considered. First, the sample was small (*n* = 5), most of whom were undergraduate psychology students, with one a master's student in social work. While this reflects the composition of the co‐researcher group involved in the guideline development project, it limits the generalisability of the findings to adolescents with different educational backgrounds, levels of research experience, or forms of involvement. As this study was qualitative and exploratory in nature, the aim was not to produce generalisable conclusions but to provide a case study of gaining an in‐depth, context‐specific understanding of adolescent co‐researchers' experiences and impacts of their involvement on them. Consequently, the transferability of the findings to other adolescent populations, research topics, or contexts may be limited and should be considered in relation to contextual similarities. This limitation mirrors challenges identified in other youth‐ and lived‐experience‐led evaluations, where findings are based on a small number of contributors' personal perspectives and therefore prioritise depth of insight over generalisability [40]. Second, there was a lack of explicit planning to explore potential negative outcomes, such as disengagement or dissatisfaction, which could have affected the comprehensiveness of the evaluation.

## 5 Conclusion

This study shows that adolescents value structured training, supportive partnerships, and autonomy in research involvement, particularly when aligned with their personal goals. These practices were associated with tangible outcomes such as skill development, co‐authorship, and strong relationships. To maximise benefits, researchers should tailor involvement processes to adolescents' needs, supported by a clear theory of change. Importantly, adolescent engagement is shaped not just by individual motivation, but also by social and institutional factors, highlighting the need for system‐level changes to enable meaningful and equitable involvement.

## Author Contributions

Conceptualization: Azza Warraitch, Ciara Wacker, Kristin Hadfield. Methodology: Ciara Wacker, Katherine Moore. Investigation: Katherine Moore. Formal analysis: Katherine Moore, Azza Warraitch. Resources: Ciara Wacker, Katherine Moore. Writing – original draft: Azza Warraitch. Writing – review and editing: Katherine Moore, Ciara Wacker, Kristin Hadfield, Azza Warraitch. All authors reviewed and approved the final manuscript.

## IRB Statement

Ethics approval for this study was obtained from the School of Psychology Research Ethics Committee at Trinity College Dublin (Application number: 3432).

## Conflicts of Interest

The authors declare no conflicts of interest.

## Data Availability

The data that support the findings of this study are available on request from the corresponding author. The data are not publicly available due to privacy or ethical restrictions.
